# Using a Scintillation Detector to Detect Partial Discharges

**DOI:** 10.3390/s19224936

**Published:** 2019-11-13

**Authors:** Łukasz Nagi, Michał Kozioł, Michał Kunicki, Daria Wotzka

**Affiliations:** Institute of Electrical Power Engineering and Renewable Energy, Opole University of Technology, Prószkowska 76 Street, 45–758 Opole, Poland; m.koziol@po.edu.pl (M.K.); m.kunicki@po.edu.pl (M.K.); d.wotzka@po.edu.pl (D.W.)

**Keywords:** radiation sensing technologies, partial discharges, scintillations, air insulation, photomultiplier

## Abstract

This article presents the possibility of using a scintillation detector to detect partial discharges (PD) and presents the results of multi-variant studies of high-energy ionizing generated by PD in air. Based on the achieved results, it was stated that despite a high sensitivity of the applied detector, the accompanying electromagnetic radiation from the visible light, UV, and high-energy ionizing radiation can be recorded by both spectroscopes and a system commonly used to detect radiation. It is also important that the scintillation detector identifies a specific location where dangerous electrical discharges and where the E-M radiation energy that accompanies PD are generated. This provides a quick and non-invasive way to detect damage in insulation at an early stage when it is not visible from the outside. In places where different radiation detectors are often used due to safety regulations, such as power plants or nuclear laboratories, it is also possible to use a scintillation detector to identify that the recorded radiation comes from damaged insulation and is not the result of a failure.

## 1. Introduction

The earlier a source of partial discharges (PD) is detected, the faster it can be reacted to, thus reducing the probability and cost of failure. PD detection is the recording of signals from phenomena associated with discharges. Early detection of electrical discharges in electrical devices and wires is an important part of diagnostics in electrical engineering. The subject area of this paper relates to high-voltage diagnostics with respect to PD. Understanding the phenomenon of electric discharges, their occurrence mechanisms, and propagation is a significant factor that helps to improve the diagnostics of energy devices. Research studies associated with electrical discharges increase the overall knowledge of this dangerous phenomenon and contribute to the development of the branch. Currently, in the high-voltage diagnostics of insulation systems, several methods are applied, which allow for the assessment and evaluation of PD. The most widely-known and commonly used research methods are the electric [1] and Ultra High Frequency (UHF) [2,3,4,5] methods, dissolved gas analysis (DGA) or Density Funcitonal Theory (DFT) [6,7] method, and the acoustic emission method [8,9]. During the occurrence of PD, air ionization and ozone production occur. Ozone testing can be effective, in itself, to assess PD levels [10]. The fact that the oxygen molecules are ionized shows the appearance of high-energy E-M radiation. Technological progress in optics enabled the development of a relatively new method for identification and localization of PD in air, with regards to the spectroscopy method [11,12]. Spectrophotometer enables for a more precise determination of the location where PD occurs. However, there is a gap in research on PD, both in the energy balance and in the phenomena related to the generation of PD. There are studies suggesting the generation of electromagnetic radiation during PD [13,14]. They can have a significant impact both on energy loss and safety, but also on the results of measurements of other values measured during PD detection. They may also have an impact on faster wear of electrical insulation in cables or on faster degradation of the cable itself. Some articles describe the stimulation to generate PD with X-ray pulses [15,16]. The discharges themselves generate X-rays. All of this leads to the destruction of the insulation. It can also cause a false interpretation of the E-M radiation source in objects such as nuclear laboratories and nuclear power plants. In such cases, it is worth knowing that the source of the registered radiation is PD. Some articles describe PD that occur in fission chambers [17]. This article and the studies presented in it fill this gap. Additionally, with regard to the influence of radiation on the conductors and their insulation.

In this paper, we consider the emission of high-energy electromagnetic radiation. The main aim is to investigate if and how much high-energy radiation is emitted by PD. Based on previous studies, it was stated that emission of high-energy ionizing radiation constitutes a significant phenomenon occurring during the generation of PD in oil.

The condition of the electric discharge is the presence of ionizing factors or free-electron sources. In gases, e.g., the air, which is a natural dielectric, light flashes and other accompanying effects, e.g., acoustic, can be observed. Other phenomena, which are not immediately visible, can be measured using many methods. Often, the discharge is determined by the phenomena resulting from the acceleration of electrons and ions by the electric field in the discharge itself.

Complete discharges may occur as a spark or electric arc. This form causes a low resistance short circuit of the electrodes. The discharge occurs between two electrodes and is characterized by low internal electrical resistance. In the area of the electric arc, the gas is highly ionized and reaches a high temperature. In the air, under atmospheric pressure and with a current flow of 1 Ampere, it is about 5000–6000 Kelvin. The ionized gas is in the form of a plasma and its temperature depends on the type of gas, its pressure, but also on the current, and the type of electrodes. A PD is a local breakdown of a small part of the electrical insulation under high-voltage stress.

The mechanism of PD and the process of breakdown using gas insulation are similar to those in ideal liquid dielectrics. Insulating liquids in real systems are often contaminated with dissolved gasses or small solids. All of this influences the process of dielectric decomposition. Air testing is an attempt to measure the energy of the radiation itself in a facility where it is easier to register. The results can be used in subsequent studies to model the phenomenon in different isolation centers or under different environmental conditions.

Scintillation detectors are widely used in medical and industrial imaging. Different scintillation materials and photomultiplier models are used in the construction. Choosing the right scintillator and photomultiplier for planned research and measurements requires determining the characteristics of the scintillation material. New materials are still being produced [18,19]. Existing solutions in different configurations are also being tested [20]. Reducing the impact of radiation on electrical equipment can be achieved by selecting the right scintillator. The most widely used scintillator is NaI(Tl). Its properties have been studied since the 1950s. Recently, its spectroscopic and luminescent properties at liquid nitrogen temperature have also been studied [21]. Apart from the scintillator, the detection systems also have detectors of light coming from the scintillator. The most commonly used are conventional photomultipliers (PMT). More and more often, however, detectors using silicon photomultiplier (SiPM) are used. The energy resolution for the NaI-SiPM detector is as good as the NaI-PMT detector [22].

## 2. Measuring Setup

**Figure 1:**
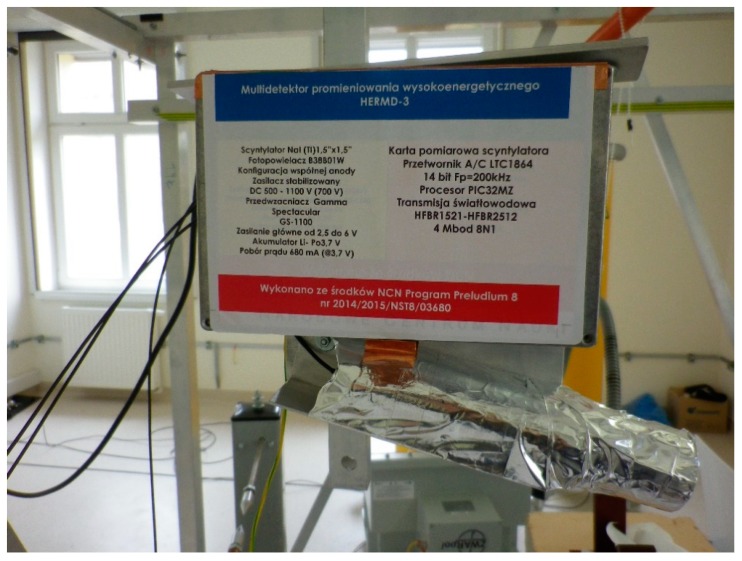
Scintillation detector based on a scintillating crystal, photomultiplier tube, and a measuring card.

The main object in the test stand is to create a system for generating full and partial electrical discharges. It is comprised of a set of PD model sources supplied by a high-voltage transformer. The transformer is controlled from a control panel, which is connected to a computer. In order to reduce interference of the electric field, a wireless driver for Xbee radio link is applied for data transmission. The component applied for electromagnetic radiation measurement was made up of a scintillation detector based on a scintillating crystal, photomultiplier tube, a measuring card (Figure 1), and a compact precise controlled 3-dimensional (CPC3D) system, which allows for a precise positioning of the detector in any point in space with an accuracy of 0.1 mm. As it was written, the radiation measurement system consisted of a scintillation detector. The detector itself can be divided into three parts. The first element is a scintillation crystal responsible for changing high-energy radiation to radiation with a different wavelength range. Another element of the detector is a photomultiplier. The last element is a measuring card. The scintillation crystal NaI(Tl) with dimensions of 1.5" × 1.5" was used in the construction of the scintillation detector. The scintillator was placed in front of the photomultiplier in a scintillation probe directed towards the radiation source. In the measurements, the Adit photomultiplier, model B38B01W with the maximum voltage between the cathode and the anode being 1500 V, was used. The photomultiplier had a system of 6 dynodes amplifying the signal. The photocathode of the photomultiplier was a semitransparent alloy called Bialkali (Sb-K-Cs) and it cooperated well with the NaI(Tl) crystal. The spectral range of the photomultiplier operation ranges from 300 nm to almost 650 nm of visible light wavelength. The photomultiplier and scintillator consisted of a scintillation probe manufactured by Ludlum. The detectors power supply voltage was 700 V, while the preamplifier was supplied by a 3.7 V lithium-polymer battery with a capacity of 13,000 mAh. The preamplifier applied was the GS-1100 PRO from Gamma Spectacular. Data recorded by the detector and its measuring card are transmitted to the computer via fiber optic cables. It is possible thanks to the use of an integrated circuit, based on the PIC32 microcontroller and HFBR1521 module. The task of the system is to receive data from the measuring card and transmit it in real-time to the computer. For this purpose, the circuit board receives data from the measuring card, then they are modulated at the input to the fiber optic link, and then demodulated and transmitted to the computer. To convert the voltage scintillation pins into a digital form, the LTC1864 analog–digital converter manufactured by Linear Technology is used. The converter is a 16-bit chip with a maximum sampling frequency of 250 kHz. The power supply current shall not exceed 0.85 mA. The driver uses the successive approximation (SAR) method using switched capacities. Communication with the host microcontroller is via the serial interface SPI. The circuit is powered by 5V.

The construction of the CPC3D is 2 × 2 × 2.5 m and is aluminum-based with an arm able to move in three dimensions. The detector is installed on the arm and is positioned next to the negative electrode during measurements, or at a specified distance from this electrode depending on a test trial. Tests were performed for the following 3 systems enabling the generation of PD, a needle–needle system, a sphere–sphere system, and a system for generating surface discharges.

At the same time, for the possibility of verifying the obtained results, the PD phenomenon was also measured using the electric method. For apparent charge measurements, the MPD600 system is used, which meets all modern standards and is contemporary. The measurement configuration consisted of a 1 nF MCC210 coupling capacitor, a 30 µF four-pole CPL542A (also used for impedance measurement), and an MPD600 as a PD analyzer.

## 3. Measurement Methodology

**Figure 2:**
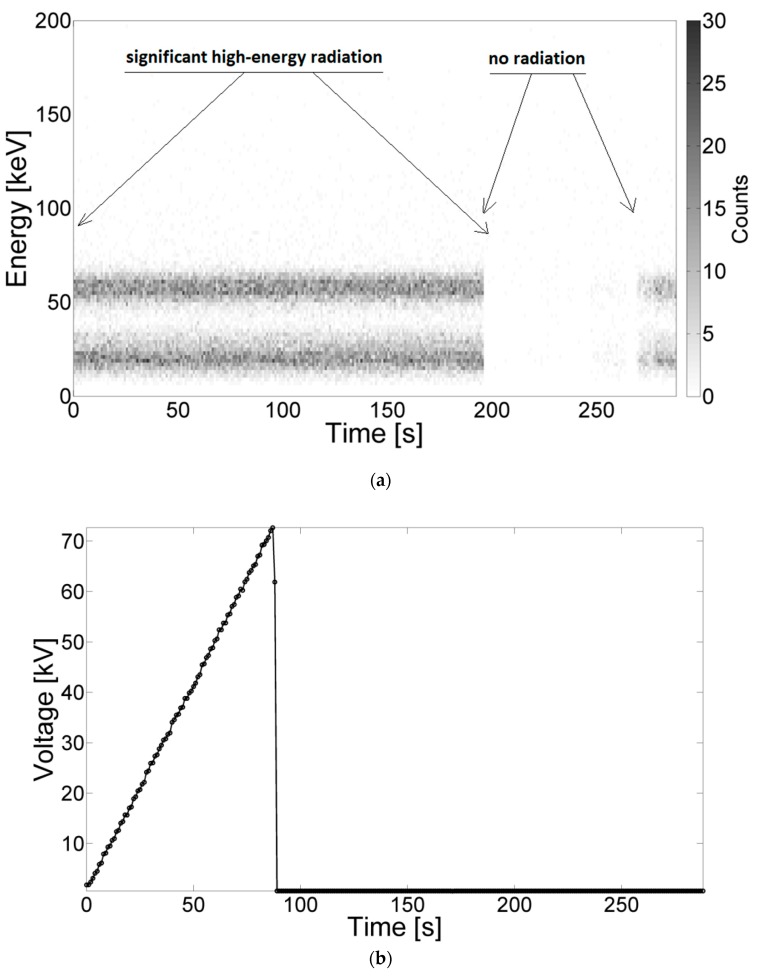
The energy spectrum of americium recorded by the detector (**a**) and a graph of increasing voltage between the electrodes during the test (**b**) until the breakdown.

Prior to the measurements, the detector was calibrated using a source of radiation, which was, in this case, americium (^241^Am). Afterward, americium was placed near the electric field generated by surface discharges. The distance between the detector and the source of discharges was 30 cm, whereas americium was positioned between the detector and the positive electrode near the detector. During the calibration procedure, supply voltage value was increased until the breakdown of the system occurred (see Figure 2b). The energy radiation spectrum was recorded during the voltage increase period, several minutes after its disappearance, and subsequently several seconds after americium was removed from the detector area. The registered signals are shown in Figure 2a. From Figure 2a, energy radiation of significant quantity (up to 30 counts) and characteristic values (about 20 keV and 50 keV characteristic for ^241^Am) can be recognized independently of the supply voltage value. After removal of the americium, no high-energy signals were detected. Both during calibration and radiation measurements, stable environmental conditions were ensured, i.e., constant temperature, humidity, and atmospheric pressure. A more detailed description of the measuring system and the calibration of the detectors can be found in [13,23]. 

As an insulating medium, air at atmospheric pressure was considered in the presented research studies. Tests were performed for 3 systems enabling generation of PD:

⚬ A needle–needle system, installed at various distances between the electrodes, changed with a step of 20 mm from 20 mm to 200 mm for each measurement trial;
⚬ A sphere–sphere system (the diameter of the spheres = 50 mm) for distances between the electrodes from 20 mm to 200 mm, increased by 20 mm for every next measurement;
⚬ A system for generating surface discharges, where a 3 mm glass plate was applied as the insulator between the electrodes.

Investigations of surface discharges were carried out for three different distances between the detector and the PD source, as well as for three different distances between the positive electrode and the glass plate (for each distance between the detector and the PD source). During the measurements, the supply voltage value was increased until the breakdown of the system occurred.

At the same time, together with radiation measurements, the electrical method was applied to determine the apparent charge of the phenomenon. The possibility to relate the results to existing and recognized methods is intended to demonstrate that radiation detection can be an important means of the non-invasive location of PD. It is also possible to verify that PD occurred during the recording of energy in the X-ray range. Examples of electrical measurement results obtained are presented in Table 1, where Q_IEC_ is the apparent charge compliant with the standard IEC 60270, Q_Peak_ is the maximum apparent charge, N is the number of counts of PD/s, I_Dis_ and P_Dis_ are the current and power of discharge, and D is the PD density. Results of measurements using electric method in application to similar electrode systems can be found in [3].

## 4. Analysis of the Results

**Figure 3:**
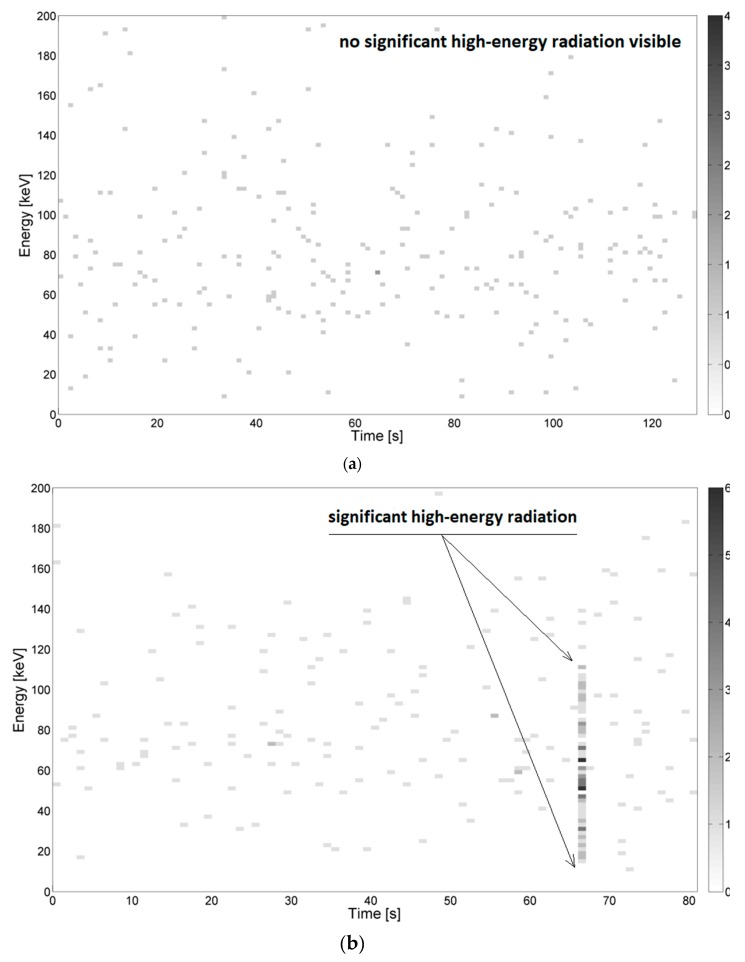

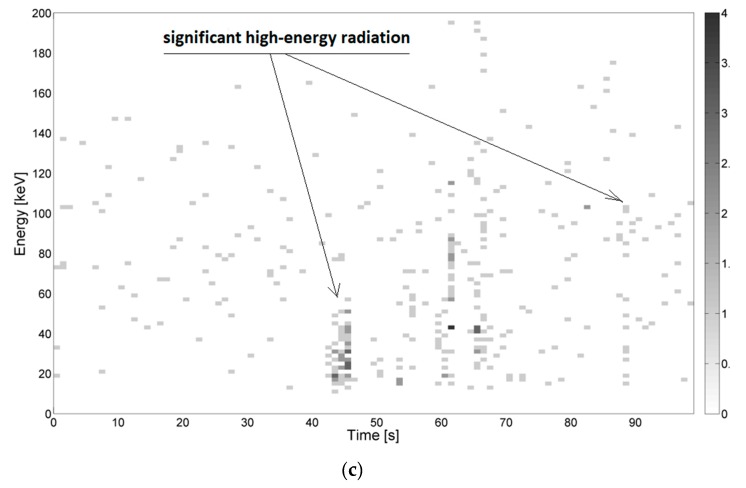
Example energy spectra of partial discharges (PD) in the needle–needle system for the following distances between the electrodes, (**a**) 100 mm, breakdown after *t =* 51 s (**b**) 180 mm, breakdown after *t =* 68 s, and (**c**) 200 mm, breakdown after *t =* 88 s. Supply voltage increased from 0 kV to 42 kV, 68 kV, and 72 kV.

**Figure 4:**
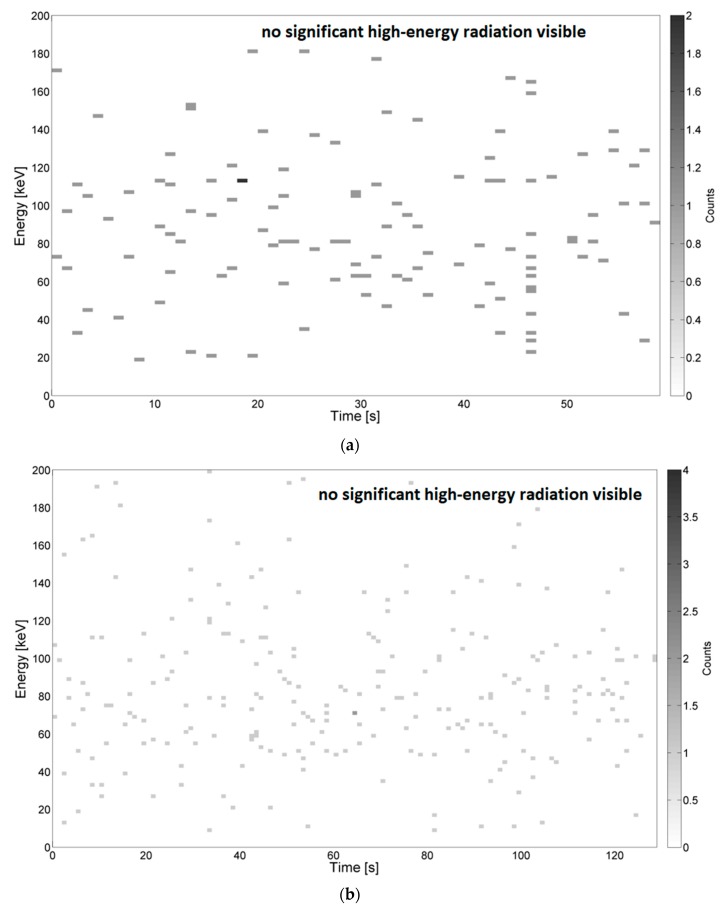

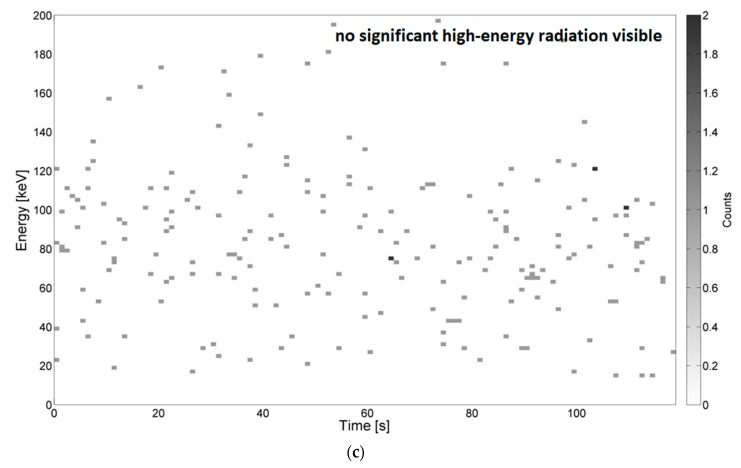
Example energy spectra of PD in the sphere–sphere system (50 mm) for the following distances between electrodes, (**a**) 100 mm, breakdown after *t* = 104 s, (**b**) 180 mm, breakdown after *t =* 102 s, and (**c**) 200 mm, breakdown after *t* = 105 s. Supply voltage increased from 0 kV to 64 kV, 83 kV, and 88 kV.

**Figure 5:**
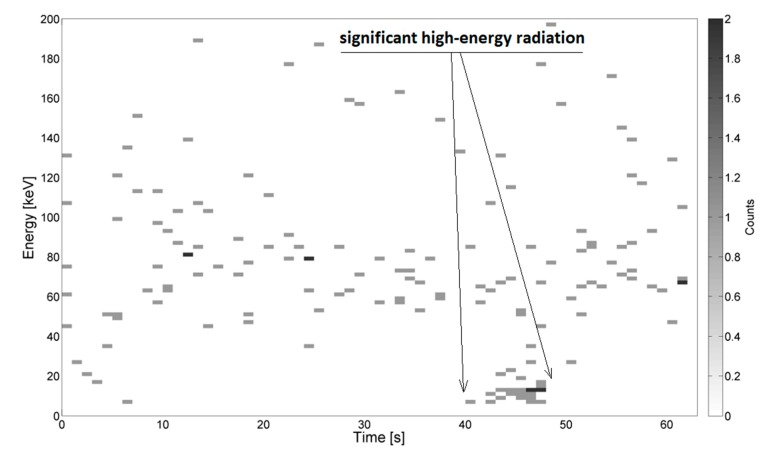
Example energy spectra of surface PD for the distance between the electrodes equal to 3 mm, supply voltage increased from 0 to 36 kV, breakdown after *t* = 49 s. The distance between the detector and the PD source was 30 mm.

**Figure 6:**
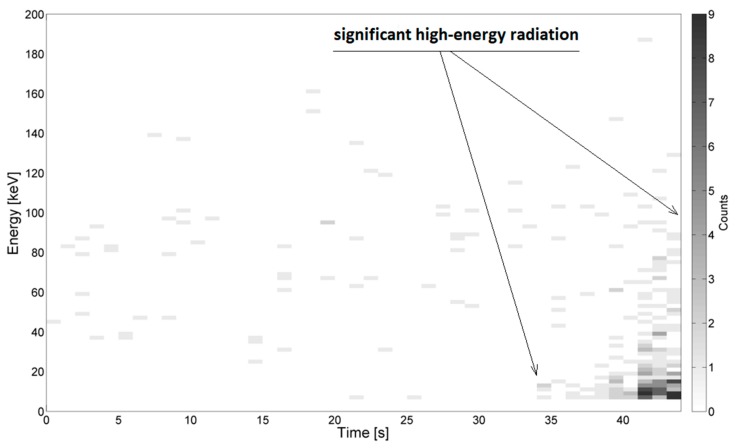
Example energy spectra of surface PD for the distance between the electrodes equal to 10 mm, supply voltage increased from 0 to 38 kV, breakdown after *t* = 45 s. The distance between the detector and the PD source was 30 mm.

Examples of the energy spectra recorded during measurements in the needle–needle and sphere–sphere systems are depicted in Figure 3 and Figure 4, respectively. Example results obtained using the system for generating surface discharges are shown in Figure 5 and Figure 6. In the presented Figures, along the ordinate, the energy value in keV is given, along the abscissa, the elapsed time is given, and the color bar is associated with the number of counts, which relates to the overall number (quantity) of energy pick of a particular energy value, registered by the device. The high-energy signals were recognized as locally increased density of counts, which were assumed to be caused by local breakdowns, thus PD occurred in the medium. Areas with increased high-energy radiation are marked with arrows. Additionally, some of the presented images do not show any significant changes in radiation, which is also marked on the respective images.

Based on the achieved analysis, no significant differences in the value of recorded energy, measured in keV, and in the quantity (count no) of generated ionization events were observed. The electromagnetic field distribution between virtual electrodes might constitute a significant factor in this case. 

Despite a high sensitivity of the applied high-energy radiation detector, no significant changes in the energy spectrum were noticed for the sphere–sphere system, whereas for the needle–needle system and the system for generating surface discharges noticeable changes were observed. 

In Figure 3a–c, energy spectra measured during generation of PD for the needle–needle system mounted at the distances of 100 mm, 180 mm, and 200 mm are presented, respectively. For lower voltage values (see Figure 3a) no significant radiation was registered. From Figure 3b, it is recognized, that right before the breakdown, which occurred after about 68 s, a sudden change of the counted number of ionizing events was noticed, accompanying an increase in energy value up to about 70 keV. In Figure 3c, locally increased concentrations of ionizing events were also observed. The increased energy values were recorded for measurement times 43–48 s, 55–57 s, 60–70 s, and 89–91 s, which correspond to the values of voltage equal to about 40 kV, 45 kV, 50–55 kV, and 72 kV respectively. In other time periods, no significant radiation of energy recorded was noticed.

In Figure 4a–c, energy spectra measured during generation of PD for the sphere–sphere system mounted at the distances of 100, 180, and 200 mm are presented, respectively. As mentioned, for all investigated distances no significant changes in the energy spectrum and no locally increased densities of high-energy events were noticed.

In Figure 5 and Figure 6, energy spectra measured during the generation of surface PD at the distance between electrodes being 3 mm (Figure 5) and 10 mm (Figure 6) are presented respectively. For both presented cases, the detector was localized at a distance of 30 mm to the PD source.

From Figure 5, it is recognized at time *t*, in the range 43–48 s, the values of energy are reaching from 10 keV to 30 keV. These signals were emitted by supply voltage ranging from 3.3 kV to about 7 kV. The most significant emission (up to 9 counts per ionizing event) was registered when the radiation detector was moved further from the PD source, at a distance equal to 30 mm. In Figure 6, it was observed that after time *t* = 38 s, while supplying the testbed with voltage values from 30 kV to 42 kV, the registered energy signals reached values from 10 keV to 40 keV. In this case, ionizing radiation was not recognized during the generation of discharges at lower voltages. 

## 5. Conclusions and Summary

The results obtained during research studies of ionizing radiation generated by PD in air were presented in this paper. The main aim was to investigate if and how much high-energy radiation is emitted by PD. Three PD generation systems were built. In a number of experiments, the influence of the distance between the high-voltage and ground electrodes, the distance between the radiation detector and PD source, and the supply voltage value on the registered radiation were estimated. Based on the achieved results the following statements were made:

- For the needle–needle spark gap system, local concentrations of the counted ionizing radiation events were noticed during the increase of supply voltage level. This was due to local breakdowns (PD) of the system tested. The mentioned indicates that the high-energy component exists and should be included as one of the phenomena accompanied by PD. This information is crucial e.g., for one who aims to investigate the balance of PD generation energy. Furthermore, the results presented suggest that the highest counted number and the highest energy was recorded right after the system breakdown;
- The energy spectra obtained during the generation of surface discharges also confirm the existence and measurability of the ionizing radiation;
- However, for the sphere–sphere spark gap system, such a characteristic spectrum was not obtained. One possible reason for it may be in the different electromagnetic field distribution between electrodes of various shapes (needle, sphere, and plate).

In general, we can state that an increase in the supply voltage did not have had any effect on the possibilities of recording ionizing radiation events. Furthermore, changes in the distance between the detector and the PD source, and changes in the distance between the electrodes, have not shown any unambiguous correlation or impact on the registered high-energy radiation. Nevertheless, it may be assumed that by supplying the setup with higher voltage values and by increasing the distance between the electrodes, the higher energy may be recorded. It may also be assumed that the distance between the detector and PD source has an effect on the number of recorded scintillation events and their energy value.

In future studies, research on the elemental composition of the electrodes, which may affect the achieved results, may be carried out. Moreover, the impact of humidity variations and other factors affecting the nature of the PD phenomenon may be interesting for investigation.

An important effect of detecting ionizing radiation and measuring its energy may be the correlation between the intensity of this radiation and the rate of degradation of the insulation of power lines. So far, the destructive effect of temperature and mechanical damage during discharges has been observed. The widening of known factors influencing the destruction of insulation is an important step towards the development of the discipline of electrical engineering and the reduction of operating costs of power equipment and power lines. 

Another important element of the presented research is the possibility of using scintillation detectors in places where E-M radiation may come from another source. Determination of the spectra from electric discharges will allow for a calm assessment of the radiation source in places such as nuclear power plants, etc.

## Figures and Tables

**Table 1:** Examples of electrical method measurement.

Parameter	Needle–Needle System	Sphere–Sphere System
Breakdown voltage	72 kV	64 kV
Q_IEC_	353.4 nC	18.57 nC
Q_Peak_	504.7 nC	28.74 nC
N	57.19 kPD/s	68.14 kPD/s
I_Dis_	1.261 mC/s	40.01 mC/s
P_Dis_	105.7 W	2.354 W
D	167.4 pC^2^/s	133.1 fC^2^/s
